# Coalescent analyses show isolation without migration in two closely related tropical orioles: the case of *Icterus graduacauda* and *Icterus chrysater*

**DOI:** 10.1002/ece3.768

**Published:** 2013-10-09

**Authors:** Nandadevi Cortés-Rodríguez, Frode Jacobsen, Blanca E Hernandez-Baños, Adolfo G Navarro-Siguenza, Jeffrey L Peters, Kevin E Omland

**Affiliations:** 1 Department of Biological Sciences, University of Maryland-Baltimore CountyBaltimore, Maryland, 21250-0001; 2 Museo de Zoología, Facultad de Ciencias, UNAMApartado Postal 70-399, México City, DF 04510, México; 3 Department of Biological Sciences, Wright State UniversityDayton, Ohio 45435

**Keywords:** coalescent, Icterus, Isolation with Migration, Isthmus of Tehuantepec, multilocus

## Abstract

The Isthmus of Tehuantepec has played an important role in shaping the avian diversity of Mexico, as well as the rest of the Western Hemisphere. It has been both a barrier and a land connector between North and South America for many groups of birds. Furthermore, climatic change over the Pleistocene has resulted in ecological fluctuations that led to periods of connection and isolation of the highlands in this area. Here we studied the divergence of two species of orioles whose distribution in the highlands is separated by the lowlands of the Isthmus of Tehuantepec: *Icterus graduacauda* (west of the Isthmus) and *Icterus chrysater* (east of the Isthmus). We sequenced multiple loci (one mitochondrial gene and six nuclear introns) and performed coalescent analyses (Isolation with Migration) to test whether their divergence resulted from prior occupancy of the ancestral area followed by a vicariant event or recent dispersal from one side or the other of this Isthmus. Results strongly indicate a vicariant event roughly 300,000 years ago in the Pleistocene followed by little or no gene flow. Both mitochondrial and nuclear genes show that the Isthmus of Tehuantepec is a strong barrier to gene flow. Thus, these two species appear to not exchange genes despite their recent divergence and the close geographic proximity of their ranges.

## Introduction

The Great American Biotic Interchange, which began around 3.5 million years ago with the completion of the Panama land bridge, resulted in numerous plants and animals moving between North and South America (Weir et al. 2009). Thus, these previously isolated fauna from temperate and tropical regions were intermixed, resulting in unique evolutionary and ecological consequences from these regions. Even though the Isthmus of Panama is the best-known portion of the land bridge, another lowland area, the Isthmus of Tehuantepec in southern Mexico, is also an important part of the connection between the Americas, the Isthmus of Tehuantepec in southern Mexico.

The Isthmus of Tehuantepec (Fig. 2) is a strip of land that constricts to a width of roughly 200 km between the Atlantic and the Pacific. Furthermore, the Oaxacan highlands to the west and the highlands of Chiapas to the east (Binford 1989; Barrier et al. 1998) are only separated by 50 km. Elevation drops from 2000+ m in the highlands on either side of the isthmus to 200 m in the lowlands, making it an important barrier to dispersal for species of higher elevation (Sullivan et al. 2000; Castoe et al. 2009; among others). The Isthmus of Tehuantepec provides a model location for studying modes of speciation as it can serve as a barrier for some species, while acting as a connector for others.

The two fundamental modes of geographic speciation are vicariance and dispersal (founder event speciation). The Isthmus of Tehuantepec may have fragmented two populations from a widespread ancestral population and isolated them for enough time that they diverge into distinctive lineages as a result of the vicariant event. On the other hand, speciation by dispersal could occur with the isthmus acting as a connector, if some individuals from either side moved across the barrier and founded a new geographically isolated population.

Following either speciation mode, the allopatric populations could experience little or no gene flow and become increasingly reproductively isolated (Coyne and Orr 2004). However, a growing body of research demonstrates that even after a population splits, gene flow can still occur in time periods following initial divergence (Hey 2005). For example, climatic oscillations (e.g., in temperature, precipitation, and sea levels) during the Pliocene or Pleistocene likely caused the Isthmus of Tehuantepec to experience alternating periods of connection and disconnection between different highland habitats (Duellman 1960; Zarza et al. 2008).

A number of studies have examined the role the Isthmus of Tehuantepec has played in the speciation of terrestrial species, including birds. The Isthmus of Tehuantepec likely acted as a barrier to dispersal, separating populations to the west and east, for montane birds such as *Chlorospingus ophthalmicus* (Sánchez-González et al. 2007), *Lampornis amethystinus* (Cortes-Rodriguez et al. 2008), and *Picoides villosus* (Klicka et al. 2011). Another study based on mitochondrial DNA examined several bird species with distributions on either side of the Isthmus and concluded that fluctuations in the climate of montane habitats had fractured the bird fauna during the late Pliocene and Pleistocene (Barber and Klicka 2010). However, other studies have found that the Isthmus of Tehuantepec has not acted as a barrier for dispersal given the lack of genetic differentiation between populations of other highland bird species such as *Lepidocolaptes affinis* (Arbeláez-Cortés et al. 2010) and *Buarremon* species (Navarro-Sigüenza et al. 2008) or lowland species such as *Icterus pustulatus*.

Traditionally, this type of biogeographic research, including both species-level phylogenetics and within-species phylogeography, has used mitochondrial DNA as a way to understand geographic variation within and between closely related species. However, mtDNA alone is often insufficient to elucidate phylogeographic patterns (Funk and Omland 2003; Peters et al. 2012a). The evolutionary history of a gene might not be congruent with the evolutionary history of a species due to lineage sorting of ancestral polymorphisms or introgressive hybridization; thus, relying on mitochondrial DNA alone can lead to incorrect conclusions about the evolutionary history of lineages (Moore 1995; Jacobsen and Omland 2012). Use of multiple independent loci provides a more complete picture of population histories because mutation, genetic drift, and natural selection operate independently in unlinked loci (Peterson and Nyári 2008; Peters et al. 2012b).

One current challenge is to study the relationship between shared genetic variation, the timing of divergence, and the geographic context of divergence along with other processes such as ongoing gene flow (Won and Hey 2005). Coalescent methods can help answer questions at the level of population divergence; indeed, these methods are best applied to within species or between recently diverged taxa (Rosenberg and Nordborg 2002). The program Isolation with Migration (IM; Hey and Nielsen 2004) uses data from unlinked genes to obtain posterior probability distributions for several demographic parameters including effective population size, migration, and time since divergence, and together, they capture many of the dynamics of the early stages of population differentiation (Hey and Machado 2003).

**Figure 1:**
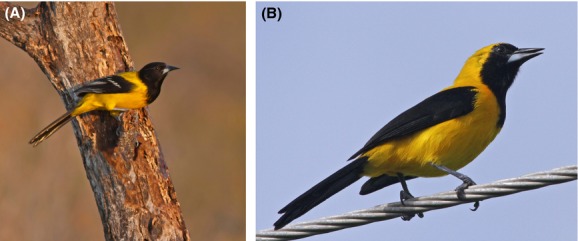
*Icterus graduacauda* (photographed by Stephen J. Pollard) and *Icterus chrysater* (photographed by Ian Davies).

Figure 1 illustrates *Icterus graduacauda* (A) and *Icterus chrysater* (B), two sister species of tropical orioles whose distributions are separated by the Isthmus of Tehuantepec, and with very different colorations. The two species are roughly 1.6% divergent in mitochondrial cytochrome *b* and ND2 (Omland et al. 1999), suggesting their split occurred less than one million years ago (Fleischer et al. 1998). These two orioles thus seem to be more closely related than any other sister species in the genus (Omland et al. 1999; Jacobsen et al. 2010). As other orioles more deeply divergent hybridize extensively, at least some gene flow between these parapatric taxa seems likely. Furthermore, these two orioles are among the few species within the genus *Icterus* that inhabit highland habitats, reaching as high as 3000 m (Jaramillo and Burke 1999). *Icterus graduacauda* inhabit highlands west of the Isthmus of Tehuantepec from Texas to Oaxaca. *Icterus chrysater* inhabits highlands east of the Isthmus of Tehuantepec from eastern Chiapas to northern South America (Howell and Webb 1995). Even though these two orioles inhabit a wide variety of vegetation types along their range, near the edges of their distribution (close to the Isthmus of Tehuantepec) *I. graduacauda* inhabits humid pine–oak forests and cloud forests above 2500 m, whereas *Icterus chrysater* inhabits arid and semiarid pine–oak forests above 1500 m (Binford 1989; Jaramillo and Burke 1999). There are no known sympatric populations of the two species, and no hybridization has been documented or suggested.

In this study, we used IM to test two alternative hypotheses. H1) the two species resulted from vicariant speciation where the Isthmus of Tehuantepec as a barrier split the widespread ancestral population into two daughter populations. H2) the two species resulted from a founder event, in which the ancestral population was located on one side of the Isthmus and a small number of individuals crossed the barrier, founding a new daughter population. The direction of colonization could either have been from west to east or from east to west. Following Hey (2005) and Peters et al. (2008), we used the splitting parameter *s* to estimate the percentage of the ancestral population that contributed to each daughter population.

## Methods

### Taxon sampling and laboratory procedures

**Figure 2:**
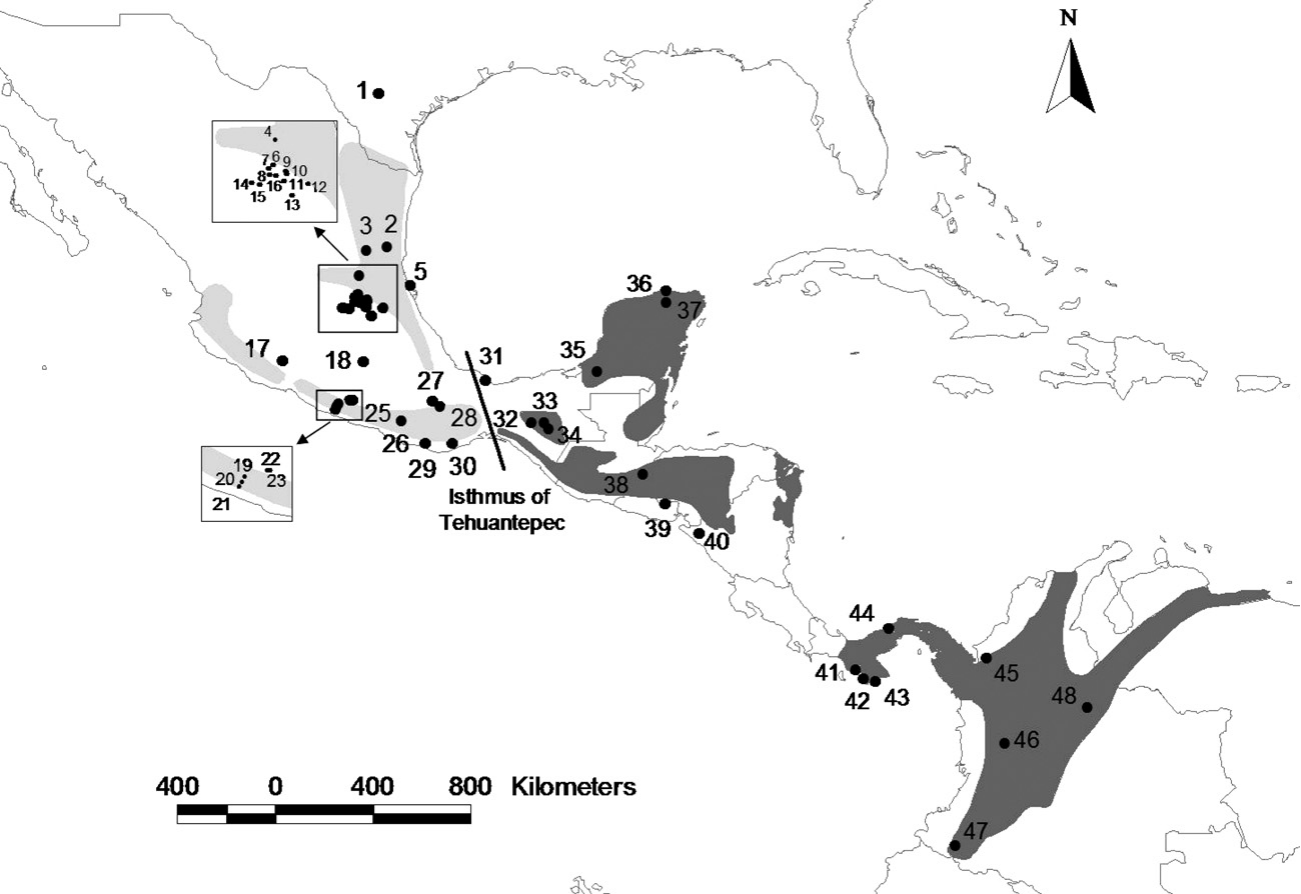
Distribution of *Icterus graduacauda* and *Icterus chrysater*. The line shows the approximate delimitation of the Isthmus of Tehuantepec following Peterson et al. (1999). Scale varies due to longitude; however, width of the Isthmus of Tehuantepec along the black line is 200 km. Each number represents a sample locality (see Supportiing information).

We obtained frozen tissues and toepads from individuals of *Icterus graduacauda* (*N* = 38) and *Icterus chrysater* (*N* = 49) collected from across each species’ range (Fig. 2). DNA was extracted from both fresh muscle and toepad samples using the DNeasy Blood and Tissue Kit (Qiagen, Valencia, CA). To improve DNA yield from toepad samples (age of the samples ranged between 1940 and 1980), we added 20 μL of dithiothreitol (DTT) to the sample in addition to 20 μL of proteinase K.

We sequenced a total of seven loci, including 344 bp of the mitochondrial control region (CR-domain I) for all individuals and six intronic regions of nuclear DNA for a subset of individuals (α-ENO, RDP2, GAPDH, TGFB2, MUSK, and SLC9, see Table 1 for details). PCR was carried out on the Gene Amp PCR System 9700 (Applied Biosystems, Foster City, CA), and products were verified on a 1% agarose gel, visualized by staining with ethidium bromide, and cleaned using QIAquick PCR purification kits (Qiagen). We sequenced these products using ABI's BigDye v.3 Terminator Cycle Sequencing Kit on the Gene Amp PCR system 2400 (Applied Biosystems, Foster City, CA). Excess dye terminators were removed by the EDTA–ethanol precipitation protocol as recommended by Applied Biosystems, and sequences were run out at University of Maryland, Baltimore County (UMBC) on an ABI 3100 DNA sequencer (Applied Biosystems). All sequences were edited and aligned using Sequencher 4.1 (Genecodes Corporation, Ann Arbor, MI).

**Table 1:** Characteristics of loci used in this study. One mitochondrial locus and six nuclear introns with the total number of base pairs, chromosome location, annealing temperature, and the primer source

Locus	Total bp	Chromosome	Annealing temperature	Primer source
Control region (CR)	344	Mitochondria	52**°**C	Kondo et al. (2004)
GADPH	283	Chromosome 1	55**°**C	Primmer et al. (2002)
TGFB	544	Chromosome 3	65**°**C	Bureš et al. (2002)
RDP2	297	Chromosome 12	56**°**C	Waltari and Edwards (2002)
ENO	252	Chromosome 21	60**°**C	Kondo et al. (2008)
MUSK	467	z-chromosome	50**°**C	Clark and Witt (2006)
SLC9	434	z-chromosome	56**°**C	Backström et al. (2006)

### Population structure and selection

We constructed haplotype networks for all loci using the median-joining algorithm in Network 4.5.1.6 (Bandelt et al. 1999). For each species, we calculated the following: nucleotide diversity (π), nucleotide polymorphisms (θ), Fu's Fs (Fu 1997), R_2_ (Ramos-Onsins and Rozas 2002), Tajima's D (Tajima 1989), and mismatch distributions (with their associated Tau and Theta) using the program DnaSP 4.0 (Rozas et al. 2003). All of the above calculations were tested; however, Tajima's D and R_2_ are the recommended statistics (Ramirez-Soriano et al. 2008). We then estimated the level of differentiation between species and populations within each species for each locus (for within- and between-species variation) using analysis of molecular variance (AMOVA) in Arlequin v3.01 (Excoffier et al. 2006). Populations within each species were established based on sample localities. We used calculations in AMOVA that took into account the number of mutations between haplotypes (Φ-statistics). In order to calculate the allelic richness for each nuclear locus, we standardized the sample sizes to the smallest sample using the software Rarefaction calculator (University of Alberta, Alberta, Canada).

### Coalescent analyses

We used coalescent analysis of six unlinked nuclear loci to study the divergence history of the sister species *I. graduacauda* and *I. chrysater* (the control region of the mtDNA was not included). The program Isolation with Migration (IM; Hey and Nielsen 2004) jointly estimates demographic parameters to obtain posterior probability distributions from unlinked genes (Hey 2007). These parameters offer the opportunity of capturing the dynamics of a population during the early stages of differentiation (Hey 2005). The model estimates the following demographic parameters scaled to the per-locus mutation rate: effective population sizes (θ_1_ = population 1, θ_2_ = population 2, and θ_A_ = ancestral population), migration rates (m_1_ and m_2_), and the time of population splitting (*t*). IM can also estimate the fraction of the ancestral population that founded each population (s and 1-s). The program IM operates under the following assumptions: (1) loci are neutral, (2) no recombination within loci, (3) free recombination between loci, (4) panmictic ancestral and descendant populations, and (5) constant migration rates over time (Hey and Nielsen 2004).

Allelic states at nuclear loci were inferred using PHASE v.2.1 (Stephens et al. 2001; Stephens and Donnelly 2003). After being phased, every locus was tested for evidence of recombination using IMgc (Woerner et al. 2007). IMgc infers recombination from violations of the four gamete rule within a sequence alignment by testing compatibility between pairs of polymorphic sites (Hudson and Kaplan 1985). Inheritance scalars were defined depending on the mode of inheritance for each locus (autosomal = 1, and Z-linked = 0.75), and the infinite-sites mutation model was used. To improve mixing of the Markov chains (to facilitate convergence), we ran multiple heated chains and kept monitoring the autocorrelation and estimates of ESS (effective sample sizes; Hey 2007). We used Isolation with Migration Analytical (IMa; Hey and Nielsen 2007) to obtain migration, effective population size (ancestral population and daughter populations) and divergence time. Because IMa does not calculate the splitting parameter *s*, we also used Isolation with Migration (IM; Hey and Nielsen 2004).

We repeated the analysis twice using the same priors but different seeds in each one of the runs. The runs included 1,000,000 generations, using 30 heated chains and sampling every 100 generations. To convert the estimated parameter into biologically informative values, we need the generation time and mutation rates. We assumed an average generation time for passerine birds of 1.7 years (Sæther et al. 2005). We used a mutation rate average of 3.6 × 10^−9^ s/s/y (Axelsson et al. 2004). These mutational rates were provided in the IM input file and used to convert the parameter estimates into biological informative values. We ran the model for 100,000,000 generations.

## Results

### Population structure, selection, and recombination

**Figure 3:**
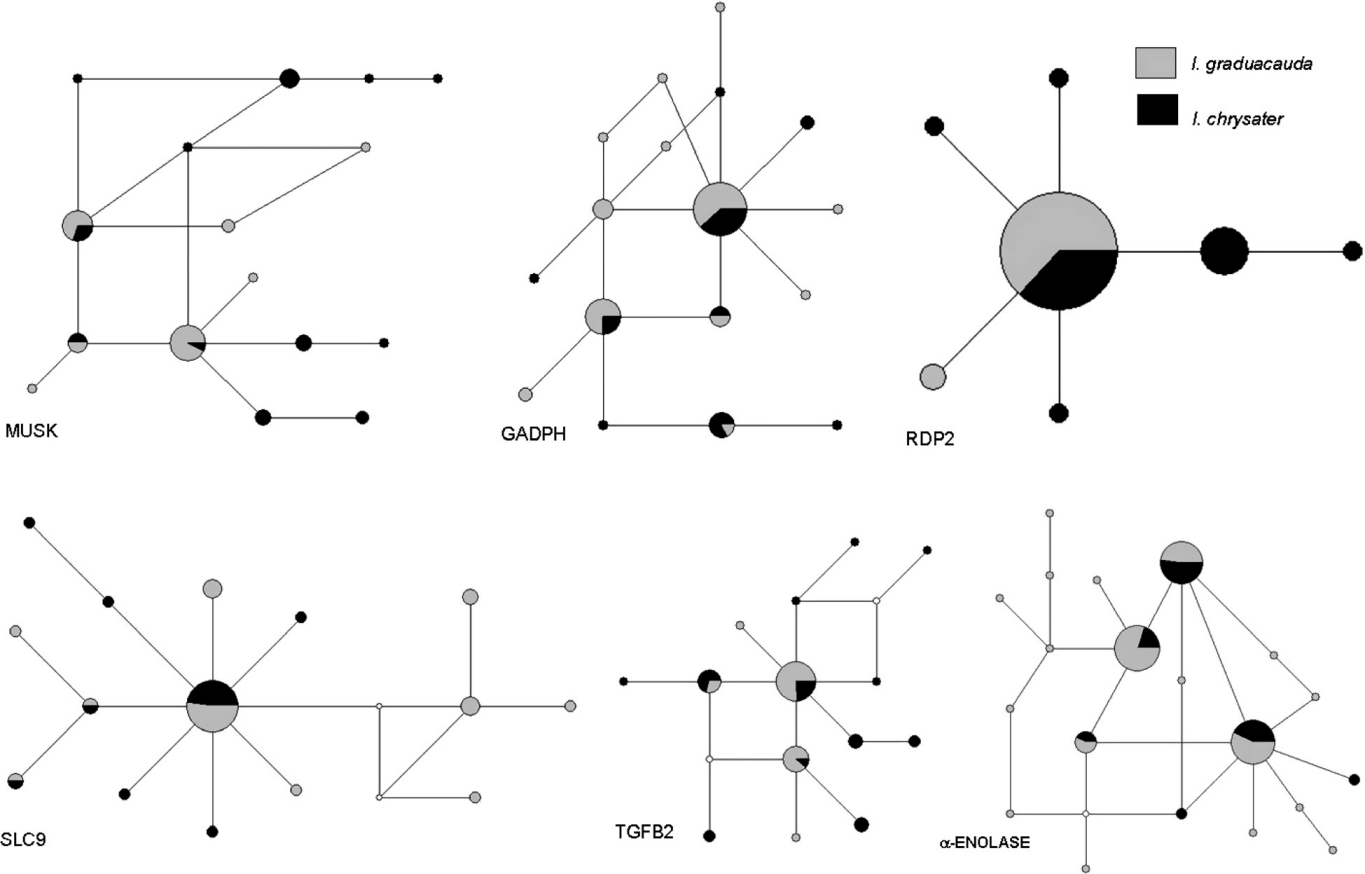
Haplotype networks of the six nuclear loci sequenced. Note that all loci show haplotype sharing between species.

Haplotype networks show allele sharing between *I. graduacauda* and *I. chrysater* across all nuclear loci (Fig. 3). Nearly all loci display a similar distinctive star-shaped pattern, with one or two shared central ancestral haplotypes surrounded by several rare haplotypes that are not shared between species, which could be the result of a recent population expansion or positive selection (Schneider and Excoffier 1999). Also apparent in the haplotype networks, all introns and the control region show high levels of haplotype diversity (except RDP2, which showed only a few haplotypes per species). Haplotype diversity, nucleotide diversity, and allelic richness vary between loci in both *I. graduacauda* and *I. chrysater* (Table 2).

**Table 2:** Estimates of haplotype diversity (including number of haplotypes), nucleotide diversity, and allelic richness (standardized to the smallest sample size for each locus) for the seven loci sequenced for *Icterus graduacauda* and *Icterus chrysater*. The highest value for nucleotide diversity each locus is shown in bold

Locus	No. of individuals (*I. graduacauda*)	No. of individuals (*I. chrysater*)	Haplotype diversity (*I. graduacauda*)	Haplotype diversity (*I. chrysater*)	Nucleotide diversity (*I. graduacauda*)	Nucleotide diversity (*I. chrysater*)	Allelic richness (*I. graduacauda*)	Allelic richness (*I. chrysater*)
CR	30	26	0.86 (15 hap)	0.46 (4 hap)	**0.0060**	0.0015	13.38 ± 0.92	4 ± 0.00
RDP2	26	24	0.14 (2 hap)	0.61 (6 hap)	0.0005	**0.0026**	1.99 ± 0.05	6 ± 0.00
SLC9	24	18	0.77 (8 hap)	0.57 (7 hap)	**0.0029**	0.0022	10.04 ± 1.32	7 ± 0.00
MUSK	27	23	0.72 (7 hap)	0.93 (12 hap)	0.0026	**0.0048**	6.63 ± 0.54	12 ± 0.00
TGFB	28	26	0.65 (6 hap)	0.91 (12 hap)	0.0015	**0.0038**	4.85 ± 0.36	12 ± 0.00
ENO	60	50	0.74 (8 hap)	0.85 (16 hap)	0.0044	**0.0069**	7.33 ± 0.73	16 ± 0.00
GADPH	42	24	0.73 (11 hap)	0.69 (7 hap)	0.0036	**0.0039**	9.43 ± 1.28	7 ± 0.00

**Figure 4:**
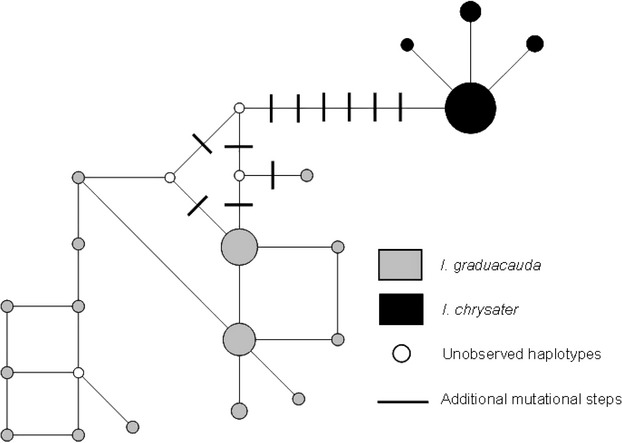
Haplotype network for the mitochondrial control region. Circles are drawn proportional to number of haplotypes (note: no haplotype sharing is observed).

The control region haplotype network (Fig. 4) shows no haplotype sharing between *I. graduacauda* and *I. chrysater*, which could be due to the rapid sorting rate of mtDNA compared with nDNA. These two groups are on average 2.6% divergent. Assuming a rough divergence rate of 4.0% per million years for control region (Fleischer et al. 1998), the mtDNA of these two species started diverging around 600,000 years ago. *I. graduacauda* had substantially higher mitochondrial haplotype and nucleotide diversity than *I. chrysater* (haplotype diversity: 0.86 vs. 0.46; nucleotide diversity: 0.0060 vs. 0.0015; see Table 2). However, nucleotide diversity between the two species varies greatly between the introns; for example, *I. graduacauda* has a higher nuclear diversity in GADPH and SLC9, whereas *I. chrysater* has a higher nuclear diversity in α-ENO, RDP2, TGFB2, and MUSK (Table 2).

The AMOVA results (Table 3) indicate high genetic variation within populations of the two species (*I. graduacauda* and *I. chrysater*) for most of the nuclear introns and with low Φ_st_ values (except for GADPH). As expected, given fixed mtDNA differences, genetic variation between species is more apparent when mtDNA is analyzed, with species explaining 84.9% of the total variation and a Φ_st_ of 0.88, which is expected given the lack of haplotype sharing between these two oriole species, as can be observed in the haplotype network. Although sample sizes are generally too low to warrant AMOVA on individual loci, all nuclear loci show low amounts of variation explained by the between-species comparison.

**Table 3:** AMOVA results and Φ_st_ value significance and percentage of variation explained between species

Locus	Φ_st_	*P*-value	% of variation between species	% of variation among populations within groups	% of variation within populations
CR	0.88	0.00000 ± 0.00000	84.91	3.38	11.71
RDP2	−0.068	0.96090 ± 0.00529	13.47	−20.31	106.84
SLC9	0.36	0.00293 ± 0.00216	4.87	31.51	63.63
MUSK	0.42	0.00000 ± 0.00000	3.69	38.48	57.83
TGFB	0.23	0.00000 ± 0.00000	2.36	21.08	76.56
ENO	0.14	0.00391 ± 0.00185	0.83	13.38	85.79
GADPH	0.60	0.00000 ± 0.00000	−6.00	66.35	39.53

Table 4 summarizes the results of the three tests of neutrality. Most loci show negative Tajima's D values (with the exception of α-ENO for *I. graduacauda* and GAPDH for *I. chrysater*). Also, Fu's Fs values are negative in both species for all loci. However, none of the loci used in this study depart significantly from neutrality (*P* > 0.05).

**Table 4:** Test for deviation from selective neutrality and constant population sizes between *Icterus graduacauda* and *Icterus chrysater*

	Tajima's D		Fu's Fs		R2	
			
Locus	*I. graduacauda*	*I. chrysater*	*I. graduacauda*	*I. chrysater*	*I. graduacauda*	*I. chrysater*
CR	−0.249	−1.04	−9.727*	−1.534**	0.115	0.092
RDP2	−0.713	−1.243	−0.317	−2.813*	0.073	0.086
SLC9	−0.739	−1.791	−3.66**	−3.955*	0.098	0.085
MUSK	−0.248	−0.017	−1.705	−5.003*	0.122	0.129
TGFB	−0.751	−0.506	−1.524	−5.538	0.06	0.106
ENO	0.096	−0.363	−2.285	−9.75**	0.11	0.093
GADPH	−1.006	0.176	−6.175**	−1.928	0.073	0.139

Significance of the observed values is indicated at 0.05 > 0.01 *P* (*) and *P* > 0.001 (**). Tajima's D and Fu's F show that the sequences are negative for the seven loci in both species.

### Coalescent analysis using Isolation with Migration

**Figure 5:**
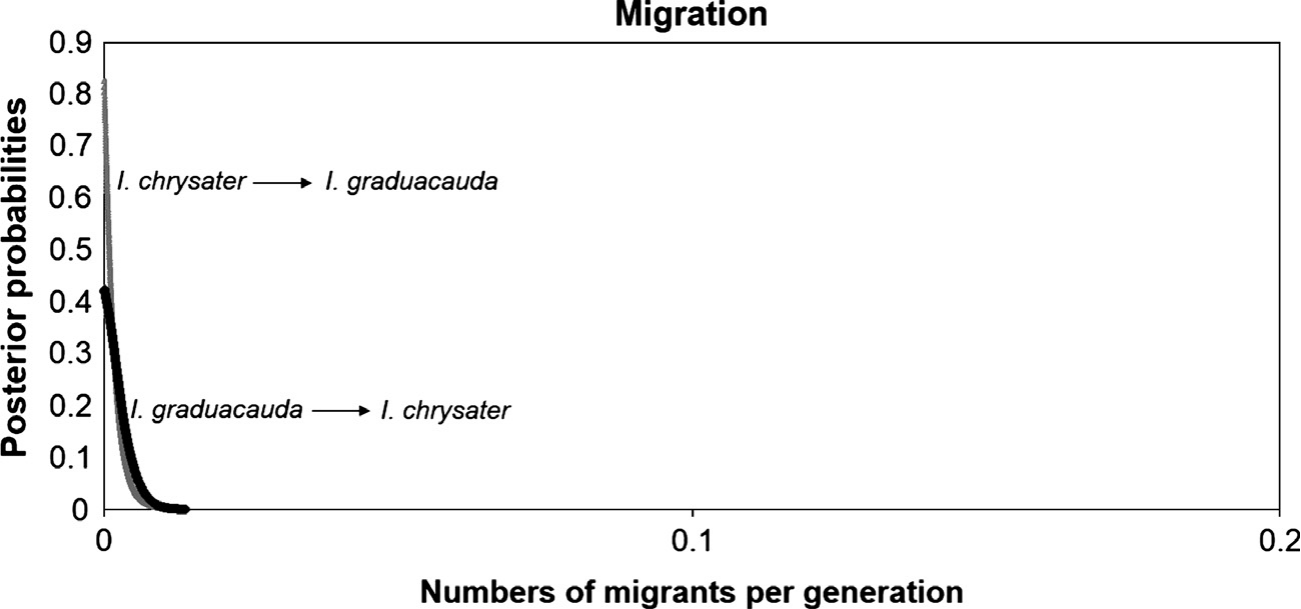
Migration probabilities from *Icterus chrysater* into *Icterus graduacauda* (gray line). And migration *Icterus graduacauda* into *Icterus chrysater* (black line) and migration (note: highest posterior probability for both directions is zero migrants per generation).

**Figure 6:**
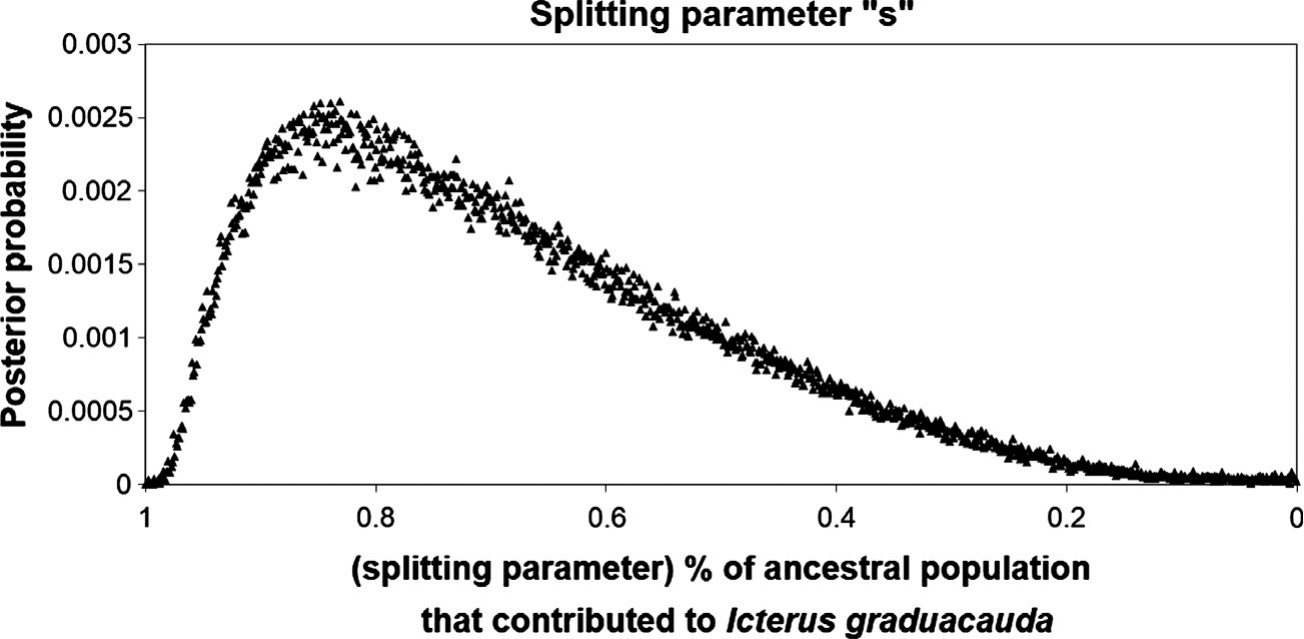
Posterior distribution of the *s* splitting parameter from the Isolation with Migration model based on the six nuDNA introns.

We found some evidence of recombination at all loci (except mtDNA) that were filtered using IMgc online. IM (Hey and Nielsen 2004) analysis of the six nuclear intron loci strongly indicates little to no gene flow between *I. graduacauda* and *I. chrysater,* with a peak estimate of migration at zero migrants per generation in both directions across the Isthmus of Tehuantepec (Fig. 5; *I. graduacauda* 95% HPD 0–0.002 and *I. chrysater* 95% HPD 0–0.004). The effective population size of *I. graduacauda* is about 300,000 individuals (95% HPD 150,000–620,000), whereas Ne for *I. chrysater* is about 600,000 individuals (95% HPD 320,000–1,200,000). The ancestral population size was estimated as 180,000 individuals (95% HPD 45,700–256,000) individuals. Also, the multilocus IM analysis suggests that *I. graduacauda* and *I. chrysater* split roughly 300,000 years ago (95%HPD 176,000–860,000). This time estimate indicates that these two species likely diverged sometime during the Pleistocene when climate change and vegetation shifts due to glacial cycles affected many other species as well (Hewitt 2004). Moreover, the splitting parameter *s* shows a peak at 0.84 signifying that 84% of the ancestral population contributed to *I. chrysater,* while 16% of the ancestral population contributed to *I. graduacauda* (Fig. 6).

## Discussion

### Vicariance across the Isthmus of Tehuantepec and no gene flow between Icterus graduacauda and Icterus chrysater

Multilocus coalescent methods can be used to test hypotheses regarding the signatures of vicariance versus founder events across geographical barriers. Also, IM results suggest high Ne estimates for both species (*I. graduacauda =* 300,000 and *I. chrysater =* 600,000), and an *s* parameter of 16% suggests that a reasonably large fraction of the ancestral population contributed to both species at the time of the split. These results thus suggest that a widespread ancestral species was divided by the Isthmus of Tehuantepec into two daughter populations. Although there is no absolute threshold value for a founder event, when using the parameter s, 5% is a reasonable rule of thumb; furthermore, our finding of 16% is considerably higher than the values observed in other studies in which researchers found evidence for a founder event scenario. For example, Hey (2005) showed that the new world population of humans was found only by less than 1% (*s* = 0.99) from the Asian population. Similarly, Peters et al. (2008) found that the North American populations of Gadwalls (*Anas strepera*) were colonized by less than 1% (*s* = 0.04) of the Eurasian population.

Surprisingly, despite the narrowness of the strip of lowland separating the distributions of *I. graduacauda* and *I. chrysater*, we found no evidence of interspecific gene flow across the Isthmus of Tehuantepec. Gene flow between the two species would have seemed likely because the distance between our samples from the Sierra Madre Oriental (*I.* graduacauda) and the Sierra Madre del Sur (*I. chrysater*) is only about 50 km. However, dispersal across this valley seems rare or nonexistent based on IM estimates of <0.005 migrants per generation from either side; if any individuals do cross, they may be unable to establish territories and/or obtain mates. These results suggest that the high levels of haplotype sharing observed across all nuclear loci are most likely due to incomplete lineage sorting and widespread sharing of ancestral polymorphism and not due to current gene flow between species (Moore 1995; Hoelzer 1997; Omland et al. 2006).

### Time since divergence between Icterus graduacauda and Icterus chrysater

According to the IM model, the most probable time of divergence between *I. graduacauda* and *I. chrysater* was estimated to be approximately 300,000 years ago (95% HPD 176,000–860,000). This estimated time of divergence between these two species is placed during the second most recent glacial maximum (Illinoian glaciation), which occurred between 130,000 and 300,000 years ago in the Pleistocene epoch (1.8 to 0.01 MYA). The Pleistocene was characterized by intense climatic oscillations around the world, with glacial and interglacial periods that changed the distribution of many species (Hewitt 2000). Pollen records show that during glacial periods, low tropical forest in southern México were displaced more than once to the south during cold periods and to the north during warmer periods (Guevara-Chumacero et al. 2010). These altitudinal and latitudinal shifts of vegetation were of crucial importance for avian evolution and speciation, as it created the opportunity for species to either move into new areas or adapt to changing conditions and in some cases go extinct (Hewitt 2000; Zink and Klicka 2006; Barrera-Guzmán et al. 2011).

### Dual aspect of the Isthmus of Tehuantepec: Barrier and connector

A variety of studies in phylogeography have focused on the Isthmus of Tehuantepec and its influence on the speciation of different taxonomic groups including birds (García-Moreno et al. 2004; Cortes-Rodriguez et al. 2008; Arbeláez-Cortés et al. 2010; Barrera-Guzman et al. 2011), mammals (Sullivan et al. 1997; Kerhoulas 2008; Guevara-Chumacero et al. 2010), amphibians (Duellman 1960), and plants *Palicourea padifolia*, (Gutiérrez-Rodríguez et al. 2011) among others. However, most of these studies lacked rigorous multilocus methods to test hypotheses of demographic processes (Rosenberg and Nordborg 2002). Furthermore, while some of these studies used statistical methods such as coalescent analysis, they were carried out using mtDNA alone, which can have limitations when accounting for stochastic processes and selection, leading to different geographic patterns for different loci (Rosenberg and Nordborg 2002; Hickerson et al. 2010; Towes and Brelsford 2012).

Our study shows how utilizing both mtDNA and nuDNA offers a more powerful and complete evolutionary history of the species of interest as it compares results from both marker types. For example, mtDNA sequences showed no intermixing of haplotypes between *I. graduacauda* and *I. chrysater,* suggesting that the two species have been isolated for sufficient time to accumulate fixed mitochondrial differences. Nuclear DNA likewise suggested a long history of divergence with little to no gene flow, despite alleles being shared between species at all loci. The shared alleles found for nuDNA are likely a result of the slower sorting rate of nuDNA relative to mtDNA and the retention of ancestral polymorphisms (Funk and Omland 2003; Towes and Brelsford 2012). The concordant signatures of complete isolation found in both marker types strengthen our conclusions about history.

The lowlands of the Isthmus of Tehuantepec have been under the influence of climatic changes and shifts of montane forests repeatedly over the last million years, creating an apparent barrier to gene flow not only for *I*. *graduacauda* and *I. chrysater* but also for other species including *Chlorospingus ophthalmicus* (Bonaccorso et al. 2008), *Lampornis amethystinus* (Cortes-Rodriguez et al. 2008), *Palicourea padifolia* (Gutiérrez-Rodríguez et al. 2011), *Peromyscus aztecus* (Sullivan et al. 1997) among others. However, it has acted as a connector for other species, such as: *Lepidocolaptes affinis* (Arbeláez-Cortés et al. 2010), *Icterus pustulatus*, and the *Buarremon* complex (Navarro-Sigüenza et al. 2008).

In conclusion, the Isthmus of Tehuantepec is the edge of the distribution for both species, and there are no historic or present reports of either of them on the other side. Not only is the Isthmus an important lowland barrier, but the narrowing of the land connection forms a funnel where only a few individuals close to the range edge are near this potential contact zone. The distributional allopatry of these two species might be explained by different natural history traits, local habitat preferences, lack of willingness to cross the lowland habitats of the Isthmus of Tehuantepec, competition with resident species, and subtle differences in diet, among others. However, it is also possible that interspecific competition for resources plays a role. Furthermore, it seems likely that differences in plumage coloration may play the crucial role in determining whether or not a few individuals that cross the lowlands obtain mates and have offspring that successfully backcross. Future work could use this interesting species pair to further explore a range of questions in biogeography and speciation.

## Supporting Information

**Table S1.** Localities of the samples we used in this study.

**Table S2.** Nuclear and mitochondrial genes that we sequenced for this study.
